# Dissecting causal networks of inflammatory factors and metabolites in heart failure: A mediation Mendelian randomization study

**DOI:** 10.1097/MD.0000000000043801

**Published:** 2025-08-08

**Authors:** Xiuyan Guan, Yanping Wang

**Affiliations:** a Department of Geratology, Cardiovascular Ward, The First Hospital of China Medical University, Shenyang, China; b Department of Interventional Radiology, The First Hospital of China Medical University, Shenyang, China.

**Keywords:** blood metabolites, heart failure, inflammatory factors, mediation analysis, Mendelian randomization, precision medicine

## Abstract

Heart failure (HF) represents a major global health burden, with complex pathophysiology involving inflammatory and metabolic pathways. However, the causal relationships among inflammatory factors, metabolites, and HF risk remain unclear. This study aims to identify inflammatory factors causally associated with HF risk, characterize metabolic alterations causally linked to HF development, and explore potential mediating roles of metabolites in inflammation-related HF pathogenesis. We conducted a comprehensive Mendelian randomization study using genetic data from FinnGen biobank (29,218 HF cases and 381,838 controls), combined with genome-wide association studies data for 91 inflammatory factors and 1400 metabolites. We performed bidirectional and mediational Mendelian randomization analyses to investigate causal relationships and potential mediating effects of metabolites in inflammation-related HF pathogenesis. We identified 9 inflammatory factors (out of 91) causally associated with HF risk, including 3 risk-promoting factors (interferon gamma: OR = 1.080, matrix metalloproteinase-1: OR = 1.081, tumor necrosis factor-beta: OR = 1.064) and 6 protective factors (CD40L receptor: OR = 0.954, DNER: OR = 0.943, IL-10: OR = 0.950, LIFR: OR = 0.911, TNFSF12: OR = 0.933, uPA: OR = 0.927). More than 13 metabolites (out of 1400) showed robust associations with HF risk, with N-methyl-2-pyridone-5-carboxamide demonstrating the strongest evidence (OR = 1.058). Further analysis revealed 23 significant inflammatory factor-metabolite pairs (out of 1000s of possible combinations), suggesting potential mechanistic pathways through which inflammatory factors influence HF development. This study establishes a comprehensive causal framework linking inflammation to HF through specific metabolic alterations, identifying novel biomarkers and potential therapeutic targets. The findings suggest that combined interventions targeting both inflammatory and metabolic pathways might offer improved strategies for HF prevention and treatment.

**Key points**

- Mendelian randomization (MR) study on exploring the inflammatory and metabolic factors of heart failure (HF) risk.
- Bidirectional and mediational MR approach, utilizing FinnGen biobank genetic data and genome-wide association studies data.
- Identifying the interplay of inflammatory factors and specific metabolites related to the risk of HF: 9 inflammatory factors (out of 91) causally associated with HF, 13 metabolites (out of 1400) robustly associated with HF, and 23 significant inflammatory factor-metabolite pairs (out of 1000s of possible combinations).

## 1. Introduction

Heart failure (HF) represents a major global public health challenge, affecting approximately 56 million people worldwide with substantial mortality and morbidity.^[1]^ Despite advances in therapeutic strategies, the 5-year survival rate remains poor at approximately 50%, with the global economic burden of HF estimated at $108 billion per year.^[2,3]^ The complex pathophysiology of HF involves multiple interconnected pathways, particularly inflammation and metabolism, which have emerged as key contributors to disease development and progression.^[4]^ Inflammation plays a crucial role in cardiac remodeling and dysfunction, with various inflammatory factors including interleukins, tumor necrosis factors (TNFs), and chemokines being implicated in HF pathogenesis.^[5]^ Recent studies have demonstrated elevated levels of specific inflammatory markers in HF patients, correlating with disease severity and adverse outcomes.^[6]^ Simultaneously, metabolic dysfunction represents another fundamental aspect of HF pathophysiology, with the failing heart exhibiting significant alterations in energy metabolism, substrate utilization, and metabolic flexibility.^[7,8]^ Recent metabolomics studies have identified numerous metabolic perturbations associated with HF, including changes in fatty acid oxidation, glucose metabolism, and amino acid metabolism, which not only reflect the compromised energetic state but may also actively contribute to disease progression.^[9–11]^

However, establishing causal relationships among these factors has been challenging due to the complex nature of HF and limitations of traditional observational studies. Mendelian randomization (MR) provides a powerful approach for investigating causal relationships using genetic variants as instrumental variables, thereby minimizing confounding and reverse causation.^[12]^ While recent MR studies have successfully identified various causal risk factors for HF, no comprehensive analysis has examined the complex relationships among inflammatory factors, metabolites, and HF risk, particularly regarding potential mediating effects of metabolism in inflammation-related cardiac dysfunction.^[13–15]^ This study aims to address these knowledge gaps through a systematic investigation using bidirectional MR analysis to: (1) identify inflammatory factors causally associated with HF risk; (2) characterize metabolic alterations causally linked to HF development; and (3) explore potential mediating roles of metabolites in inflammation-related HF pathogenesis. The findings could have significant implications for HF prevention and treatment by identifying novel therapeutic targets and biomarkers, while elucidating the mediating effects of metabolism in inflammation-related cardiac dysfunction could inform the development of more effective combination therapies targeting multiple pathological pathways.

## 2. Methods

### 2.1. Data sources

This study integrated 3 large-scale genomic databases of European ancestry to ensure data heterogeneity and bias remain within reasonable ranges at same time. For the primary outcome, we utilized HF data from FinnGen biobank R10 version (finngen_R10_I9_HEARTFAIL_ALLCAUSE), including 29,218 cases and 381,838 controls. The exposure data comprised 2 genome-wide association studies (GWAS) datasets: (1) 1400 serum metabolites (GWAS Catalog ID: GCST90199621-GCST90201020) and (2) 91 circulating inflammatory factors from the University of Cambridge (GWAS Catalog ID: GCST90274758-GCST90274848).

### 2.2. MR analysis

This study employed two-sample MR to investigate the causal mechanism of inflammatory factors affecting HF through metabolic mediators. For instrument variable selection, we set the association threshold between single nucleotide polymorphisms (SNPs) and exposure factors at *P* < 1 × 10⁻⁵, and performed linkage disequilibrium analysis using PLINK 1.9 software (www.cog-genomics.org/plink2/) based on the 1000 Genomes Project European population reference dataset (https://www.internationalgenome.org/sample_collection_principles). We set the physical distance threshold at 10,000 kb and linkage disequilibrium coefficient *R*² threshold at 0.001 to ensure independent instrumental variables.^[16–18]^ The strength of instrumental variables was evaluated using F-statistics (F > 10) to control for weak instrument bias.^[19]^

The causal relationship verification was conducted in 4 steps: first, evaluating the overall causal effect of inflammatory factors on HF; second, analyzing the causal relationship between metabolites and HF; third, verifying the influence of inflammatory factors on metabolite levels; and finally, excluding reverse causation from HF to inflammatory factors through reverse MR analysis. In each step, we employed the inverse variance weighted (IVW) method for primary causal effect estimation, supplemented by sensitivity analyses including MR-Egger regression, weighted median, and mode-based methods.^[20–23]^

To assess result robustness, we implemented a series of tests, including evaluating horizontal pleiotropy using MR-PRESSO, assessing effect estimate heterogeneity through Cochran Q test, and evaluating directional pleiotropy using MR-Egger intercept test.^[24,25]^ Additionally, leave-one-out cross-validation was performed to evaluate the influence of individual SNPs on the overall effect.^[26]^ For significant causal relationships identified, comprehensive evaluation was conducted using various visualization methods including scatter plots, forest plots, and funnel plots.

For mechanism exploration, we designed a metabolite-based mediation analysis strategy, integrating results from the above 4 steps to evaluate the role of metabolites in the inflammation–HF pathway. The analysis employed conditional analysis methods to control for potential confounding factors, calculated confidence intervals for mediation effects using Bootstrap method (1000 repetitions), and conducted dose–response relationship analysis for significant mediation effects to verify causal relationship reliability. For reverse MR analysis, we obtained SNPs significantly associated with HF from the FinnGen database as instrumental variables and employed the same analytical process to evaluate potential reverse causation. The study was conducted following the Strengthening the Reporting of Observational Studies in Epidemiology using MR (STROBE-MR) guidelines.^[27]^

A supplementary table (Table S1, Supplemental Digital Content, https://links.lww.com/MD/P622) summarizing the MR assumptions and how each was tested in our study is provided.

### 2.3. Statistical analysis

All statistical analyses were performed in R software (version 4.4.1) environment (available from URL: https://www.r-project.org). MR analyses were primarily executed using the TwoSampleMR package (available from URL: https://elifesciences.org/articles/34408) for instrument variable screening, effect estimation, and sensitivity analyses, while the ieugwasr package (available from URL: https://github.com/MRCIEU/ieugwasr) was used for SNP data extraction and linkage disequilibrium analysis. In forward causation analysis, we utilized the MRInstruments package (available from URL: https://github.com/MRCIEU/MRInstruments) to obtain additional instrument variable information, and employed dplyr (available from URL: https://github.com/tidyverse/dplyr) and plyr packages (available from URL: https://github.com/hadley/plyr) for data cleaning and transformation. The reverse MR analysis workflow primarily relied on the tidyverse series packages (available from URL: https://github.com/tidyverse/tidyverse) for data processing and transformation, utilizing the purrr package (available from URL: https://github.com/tidyverse/purrr) for functional programming to enhance analysis efficiency.

Data preprocessing and integration were accomplished using the data.table package to improve large-scale genetic data processing efficiency. Study results visualization was primarily implemented through the ggplot2 package (available from URL: https://ggplot2.tidyverse.org), including the creation of scatter plots, forest plots, and funnel plots. Considering the large number of comparisons in our study (91 inflammatory factors, 1400 metabolites, and their interactions), we implemented a comprehensive false discovery rate (FDR) correction strategy. For each analysis group (inflammatory factors–HF associations, metabolites–HF associations, and inflammatory factors–metabolites associations), we applied the Benjamini–Hochberg procedure separately to control the FDR at 0.05. All reported significant associations in our final analyses met this threshold of FDR-corrected *P* < .05, ensuring that our findings are robust against multiple testing concerns.

## 3. Results

### 3.1. Causal associations between inflammatory factors and HF

**Figure 1.**
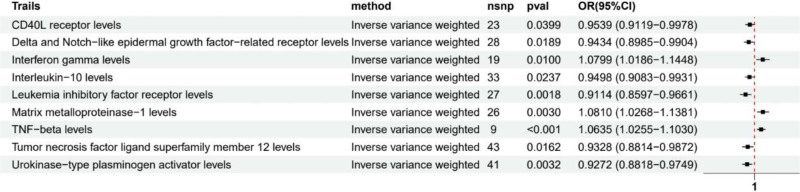
Causal associations between 9 inflammatory factors and heart failure risk.

Using genetic instruments derived from large-scale GWAS data, we conducted two-sample MR analyses to investigate the causal relationships between 91 inflammatory factors and HF risk. After applying our predefined selection criteria, 9 inflammatory factors showed significant causal associations with HF (Fig. 1, *P* < .05).

Our primary IVW analysis revealed that higher genetically predicted levels of 3 inflammatory factors were associated with increased risk of HF: interferon gamma (OR = 1.080, 95% CI: 1.019–1.145, *P* = .010), matrix metalloproteinase-1 (OR = 1.081, 95% CI: 1.027–1.138, *P* = .003), and TNF-beta (OR = 1.064, 95% CI: 1.026–1.103, *P* < .001). Conversely, 6 inflammatory factors showed protective effects against HF: CD40L receptor (OR = 0.954, 95% CI: 0.912–0.998, *P* = .040), Delta and Notch-like epidermal growth factor-related receptor (OR = 0.943, 95% CI: 0.899–0.990, *P* = .019), interleukin-10 (OR = 0.950, 95% CI: 0.908–0.993, *P* = .024), leukemia inhibitory factor receptor (OR = 0.911, 95% CI: 0.860–0.966, *P* = .002), tumor necrosis factor ligand superfamily member 12 (OR = 0.933, 95% CI: 0.881–0.987, *P* = .016), and urokinase-type plasminogen activator (OR = 0.927, 95% CI: 0.882–0.975, *P* = .003).

**Figure 2.**
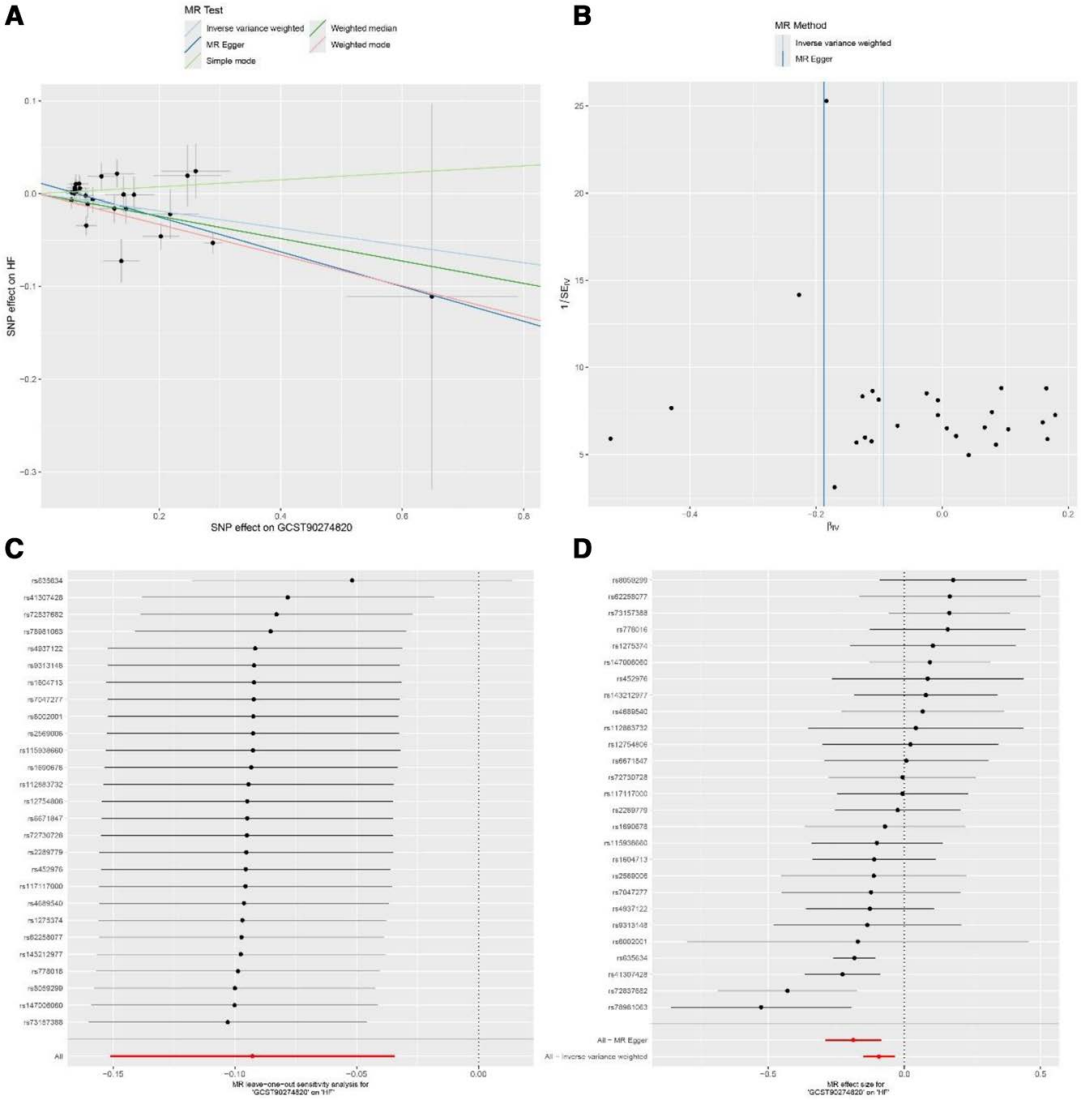
Sensitivity analyses for the association between leukemia inhibitory factor receptor and heart failure (HF). (A) Scatter plot showing the relationship between single nucleotide polymorphisms (SNP) effects on leukemia inhibitory factor receptor (x-axis) and HF (y-axis), with regression lines representing different Mendelian randomization (MR) methods. (B) Funnel plot displaying the relationship between the precision and magnitude of causal estimates for individual SNPs. (C) Leave-one-out analysis showing the stability of causal estimates when excluding each SNP in turn. (D) Forest plot showing individual SNP effects and their 95% confidence intervals.

**Figure 3.**
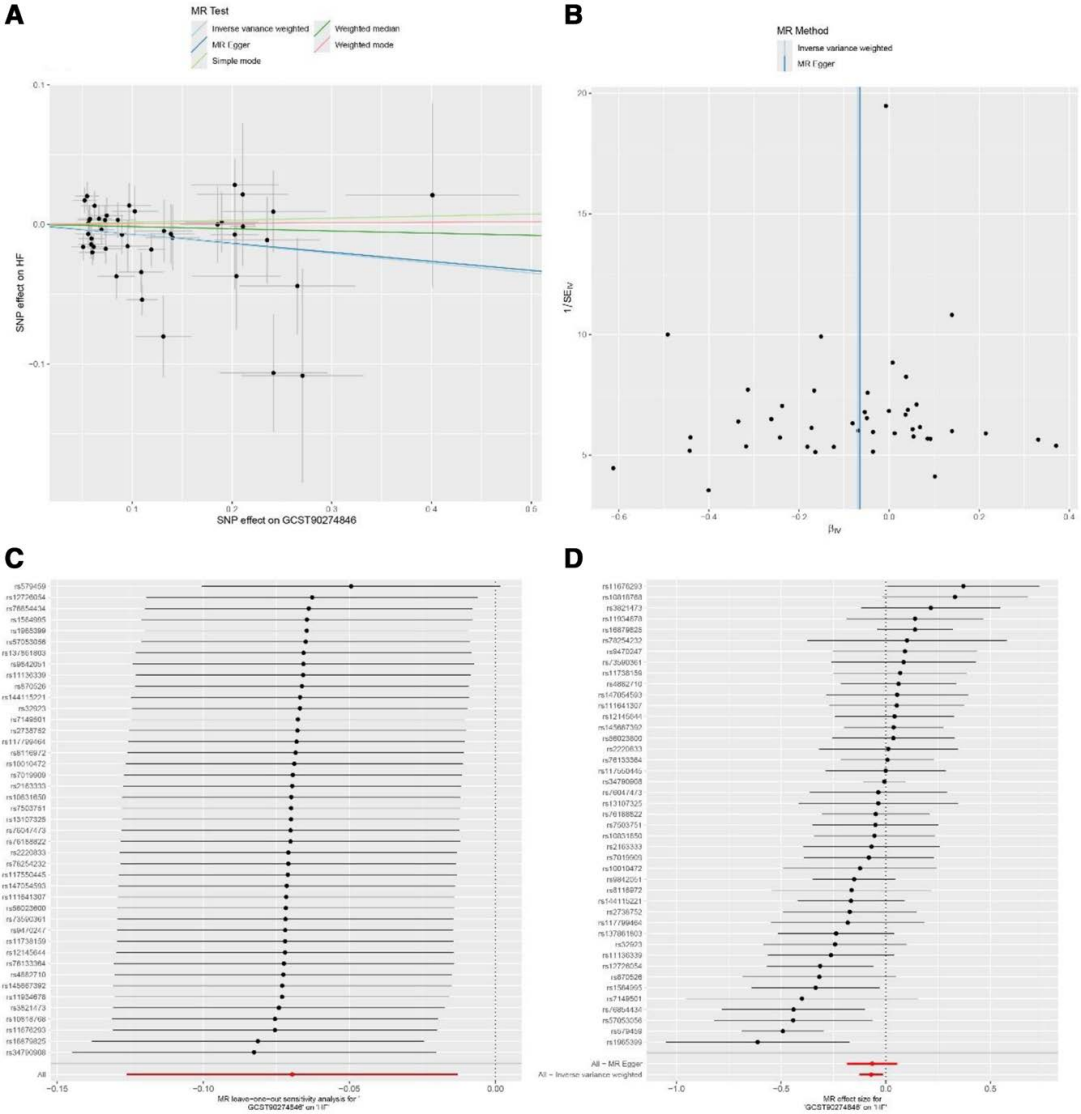
Sensitivity analyses for the association between tumor necrosis factor ligand superfamily member 12 and heart failure (HF). (A) Scatter plot showing the relationship between single nucleotide polymorphisms (SNP) effects on leukemia inhibitory factor receptor (x-axis) and HF (y-axis), with regression lines representing different Mendelian randomization (MR) methods. (B) Funnel plot displaying the relationship between the precision and magnitude of causal estimates for individual SNPs. (C) Leave-one-out analysis showing the stability of causal estimates when excluding each SNP in turn. (D) Forest plot showing individual SNP effects and their 95% confidence intervals.

Sensitivity analyses were performed to assess the robustness of these findings. The majority of identified associations remained consistent across different MR methods (MR-Egger, weighted median, and weighted mode). However, 2 inflammatory factors showed evidence of potential violations of MR assumptions. For leukemia inhibitory factor receptor, significant heterogeneity (*P* = .006) and horizontal pleiotropy (MR-PRESSO global test *P* = .005, pleiotropy test *P* = .043) were detected (Fig. 2). Similarly, for tumor necrosis factor ligand superfamily member 12, significant heterogeneity (*P* < .001) and horizontal pleiotropy (MR-PRESSO global test *P* = .001) were observed (Fig. 3), suggesting that these results should be interpreted with caution.

Funnel plots (Figs. 2B, 3B) and leave-one-out analyses (Figs. 2C, 3C) confirmed these associations were not dependent on the influence of individual SNPs. Multiple MR methods (weighted median, weighted mode, and MR-Egger regression) yielded generally consistent effect directions as shown in scatter plots (Figs. 2A, 3A) and single SNP forest plots (Figs. 2D, 3D), although statistical significance levels varied. These results suggest these inflammatory factors may play causal roles in gastric cancer development and progression.

**Figure 4.**
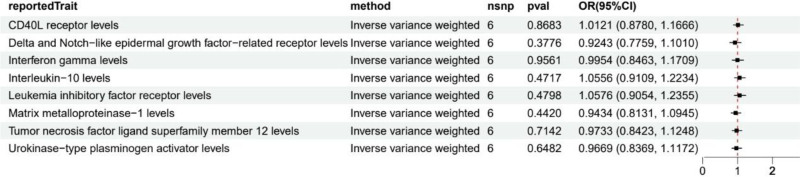
Reverse Mendelian randomization analysis results.

Additionally, to investigate whether HF might have reverse effects on circulating inflammatory factor levels, we conducted reverse MR analysis (Fig. 4). Results showed no significant causal relationships between HF and the 9 significantly associated circulating inflammatory factors.

### 3.2. Causal relationship analysis between metabolites and HF

**Figure 5.**
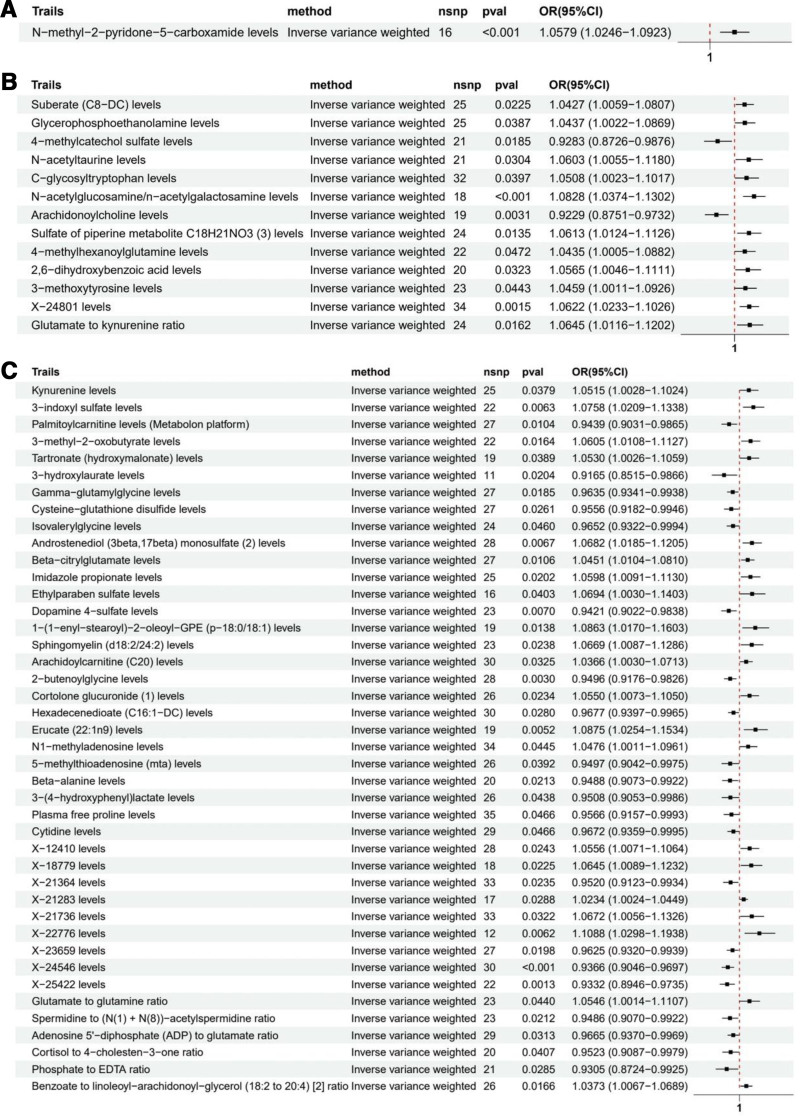
Forest plot of Mendelian randomization (MR) analysis for metabolites with strong evidence and heart failure (HF).

The MR analysis identified several metabolites significantly associated with HF risk. The strongest evidence was found for N-methyl-2-pyridone-5-carboxamide (OR = 1.058, 95% CI: 1.025–1.092, *P* < .001), which showed consistent associations across all 4 MR methods (Fig. 5A). Thirteen metabolites showed robust associations supported by at least 2 MR methods (Fig. 5B): suberate (OR = 1.043, 95% CI: 1.006–1.081), glycerophosphoethanolamine (OR = 1.044, 95% CI: 1.002–1.087), 4-methylcatechol sulfate (OR = 0.928, 95% CI: 0.873–0.988), N-acetyltaurine (OR = 1.060, 95% CI: 1.006–1.118), C-glycosyltryptophan (OR = 1.051, 95% CI: 1.002–1.102), N-acetylglucosamine/n-acetylgalactosamine (OR = 1.083, 95% CI: 1.037–1.130), arachidonoylcholine (OR = 0.923, 95% CI: 0.875–0.973), sulfate of piperine metabolite C18H21NO3 (OR = 1.061, 95% CI: 1.012–1.113), 4-methylhexanoylglutamine (OR = 1.044, 95% CI: 1.001–1.088), 2,6-dihydroxybenzoic acid (OR = 1.057, 95% CI: 1.005–1.111), 3-methoxytyrosine (OR = 1.046, 95% CI: 1.001–1.093), X-24801 (an uncharacterized metabolite in the lipid pathway, OR = 1.062, 95% CI: 1.023–1.103), and glutamate to kynurenine ratio (OR = 1.065, 95% CI: 1.012–1.120). Several other metabolites showed nominal significance in IVW analysis only (Fig. 5C).

Sensitivity analyses revealed potential heterogeneity for several metabolites based on Q test *P* values: tartronate (*P* = .029), 1-(1-enyl-stearoyl)-2-oleoyl-GPE (*P* = .044, MR-PRESSO *P* = .043), hexadecenedioate (*P* = .050), and X-21736 levels (*P* = .002, MR-PRESSO *P* = .007). The significant Q statistics and MR-PRESSO global test results suggest that the genetic variants used as instruments for these metabolites may have heterogeneous effects on HF risk. Additionally, MR-Egger intercept test suggested potential horizontal pleiotropy for cysteine-glutathione disulfide (Egger intercept *P* = .045), indicating that the genetic variants might affect HF through pathways independent of this metabolite. Given these potential violations of MR assumptions, these specific metabolites were excluded from subsequent analyses to ensure the robustness of our findings.

### 3.3. Causal relationship between circulating inflammatory factors and metabolites

**Figure 6.**
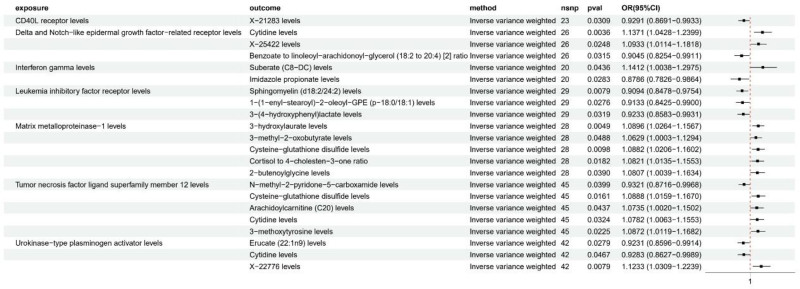
Forest plot of Mendelian randomization (MR) analysis for the causal effect of inflammatory factors on metabolites.

To further explore the potential biological mechanisms, we conducted a two-sample MR analysis to assess the causal relationships between inflammatory proteins and metabolites. After excluding metabolites that did not pass sensitivity analyses, we identified 23 significant protein-metabolite pairs involving 7 inflammatory proteins and 20 unique metabolites (Fig. 6).

CD40L receptor showed a negative causal effect on X-21283 levels (an uncharacterized metabolite in the amino acid pathway, OR = 0.929, 95% CI: 0.869–0.993). Delta and Notch-like epidermal growth factor-related receptor demonstrated causal relationships with 3 metabolites, including positive associations with cytidine (OR = 1.137, 95% CI: 1.043–1.240) and X-25422 levels (an unidentified metabolite in the lipid metabolism pathway, OR = 1.093, 95% CI: 1.011–1.182), and a negative association with benzoate to linoleoyl-arachidonoyl-glycerol ratio (OR = 0.905, 95% CI: 0.825–0.991).

Interferon gamma causally influenced 2 metabolites: suberate (OR = 1.141, 95% CI: 1.004–1.298) and imidazole propionate (OR = 0.879, 95% CI: 0.783–0.986). Leukemia inhibitory factor receptor showed negative causal effects on 3 metabolites: sphingomyelin (d18:2/24:2) (OR = 0.909, 95% CI: 0.848–0.975), 3-(4-hydroxyphenyl)lactate (OR = 0.923, 95% CI: 0.858–0.993), and 1-(1-enyl-stearoyl)-2-oleoyl-GPE (OR = 0.913, 95% CI: 0.843–0.990).

Matrix metalloproteinase-1 demonstrated positive causal relationships with 5 metabolites, including 3-hydroxylaurate (OR = 1.090, 95% CI: 1.026–1.157), 3-methyl-2-oxobutyrate (OR = 1.063, 95% CI: 1.000–1.129), cysteine-glutathione disulfide (OR = 1.088, 95% CI: 1.021–1.160), cortisol to 4-cholesten-3-one ratio (OR = 1.082, 95% CI: 1.014–1.155), and 2-butenoylglycine (OR = 1.081, 95% CI: 1.004–1.163).

Tumor necrosis factor ligand superfamily member 12 was causally associated with 5 metabolites, showing negative effects on N-methyl-2-pyridone-5-carboxamide (OR = 0.932, 95% CI: 0.872–0.997) and positive effects on cysteine-glutathione disulfide (OR = 1.089, 95% CI: 1.016–1.167), arachidoylcarnitine (OR = 1.074, 95% CI: 1.002–1.150), cytidine (OR = 1.078, 95% CI: 1.006–1.155), and 3-methoxytyrosine (OR = 1.087, 95% CI: 1.012–1.168).

Finally, urokinase-type plasminogen activator showed causal relationships with 3 metabolites: negative associations with erucate (OR = 0.923, 95% CI: 0.860–0.991) and cytidine (OR = 0.928, 95% CI: 0.863–0.999), and a positive association with X-22776 (an uncharacterized metabolite related to xenobiotic metabolism, OR = 1.123, 95% CI: 1.031–1.224).

These findings suggested complex interactions between inflammatory proteins and metabolites in the context of HF development, providing insights into potential mechanistic pathways.

## 4. Discussion

### 4.1. Principal findings

In this comprehensive MR study, we identified several key findings that provide novel insights into the complex relationships between inflammatory factors, metabolites, and HF. First, among 91 inflammatory factors examined, we found robust causal associations between 9 inflammatory factors and HF risk. Conversely, 6 inflammatory factors demonstrated protective effects. Importantly, our reverse MR analysis found no evidence of reverse causation, suggesting these inflammatory factors are likely upstream drivers rather than consequences of HF.

Second, our metabolomic analysis revealed significant associations between specific metabolites and HF risk. The most compelling evidence was found for N-methyl-2-pyridone-5-carboxamide (OR = 1.058), with consistent associations across multiple MR methods. Additionally, 13 other metabolites showed robust associations supported by at least 2 MR methods, including compounds involved in various biological processes such as amino acid metabolism (3-methoxytyrosine), energy metabolism (suberate), and lipid metabolism (arachidonoylcholine).

Third, and perhaps most importantly, our study uncovered a complex network of causal relationships between inflammatory factors and metabolites, identifying 23 significant protein-metabolite pairs involving 7 inflammatory proteins and 20 unique metabolites. These relationships suggest potential mechanistic pathways through which inflammatory factors might influence HF development.

These findings collectively establish a comprehensive framework linking inflammation to HF through specific metabolic alterations, providing new insights into the biological mechanisms underlying HF development and suggesting potential therapeutic targets.

### 4.2. Novel findings and biological mechanisms

#### 4.2.1. Protective inflammatory factors and their mechanisms

Among the protective factors, CD40L receptor (OR = 0.954) emerged as a significant protective factor against HF. Previous studies have shown that CD40L signaling plays a crucial role in cardiovascular homeostasis.^[28]^ Our findings extend this knowledge by demonstrating CD40L receptor’s negative causal effect on X-21283 levels, suggesting a potential metabolic pathway through which it may exert its cardioprotective effects. The CD40L system has been previously implicated in regulating cardiac remodeling and fibrosis,^[29]^ and our results provide new mechanistic insights into these protective effects.

Delta and Notch-like epidermal growth factor-related receptor (OR = 0.943) showed protective effects through multiple metabolic pathways. Its positive associations with cytidine and X-25422 levels, coupled with its negative association with benzoate to linoleoyl-arachidonoyl-glycerol ratio, suggest involvement in lipid metabolism and energy homeostasis. These findings align with previous research showing the importance of Notch signaling in cardiac repair and regeneration.^[30]^

Interleukin-10 (OR = 0.950) has been well-documented as an anti-inflammatory cytokine,^[31]^ and our findings provide new evidence for its protective role in HF. The protective effect of leukemia inhibitory factor receptor (OR = 0.911) is particularly interesting, as it showed negative causal effects on multiple metabolites including sphingomyelin and 3-(4-hydroxyphenyl) lactate. It suggests its involvement in both lipid metabolism and oxidative stress pathways.^[32–34]^

#### 4.2.2. Risk-promoting inflammatory factors and their mechanisms

Interferon gamma (OR = 1.080) emerged as a significant risk factor for HF, with our study revealing novel metabolic mechanisms. Its positive association with suberate levels (OR = 1.141) and negative association with imidazole propionate (OR = 0.879) suggest involvement in energy metabolism and amino acid processing. These findings complement previous research showing IFNγ’s role in cardiac inflammation and fibrosis.^[13,35]^

Matrix metalloproteinase-1 (OR = 1.081) demonstrated the most extensive metabolic network, with positive causal relationships with 5 metabolites. Its association with cysteine-glutathione disulfide (OR = 1.088) suggests involvement in oxidative stress pathways,^[36]^ while its effect on the cortisol to 4-cholesten-3-one ratio (OR = 1.082) indicates potential interactions with steroid metabolism. These findings provide new insights into how MMP-1 might promote cardiac remodeling beyond its known role in extracellular matrix degradation.^[15,37–39]^

TNF-beta (OR = 1.064), also known as lymphotoxin-alpha (LTalpha), has been previously implicated in HF progression,^[40]^ but our study reveals new metabolic pathways through which it may exert its effects. The tumor necrosis factor ligand superfamily member 12’s complex metabolic interactions, including effects on N-methyl-2-pyridone-5-carboxamide and cysteine-glutathione disulfide, suggest multiple mechanisms of action in HF development.

The intricate network of relationships we uncovered between inflammatory factors and metabolites suggests that these proteins influence HF risk through multiple, often overlapping metabolic pathways. For instance, several inflammatory factors showed connections to oxidative stress-related metabolites, suggesting this might be a common pathway in HF development.^[41]^ Additionally, the involvement of multiple lipid-related metabolites across different inflammatory factors highlights the importance of lipid metabolism in HF pathogenesis.^[33]^

The mechanistic pathways linking inflammation, metabolism, and HF pathogenesis revealed by our findings involve several key biological processes. The convergence of multiple inflammatory factors (MMP-1 and TNFSF12) on oxidative stress markers like cysteine-glutathione disulfide suggests that oxidative stress serves as a central hub in HF development. The interferon gamma-suberate relationship indicates direct inflammatory modulation of mitochondrial energy metabolism, while the strong association of N-methyl-2-pyridone-5-carboxamide with HF points to disrupted NAD + homeostasis. Additionally, the protective factors’ influence on lipid metabolites (e.g., LIFR on sphingomyelin) highlights the importance of membrane integrity and cellular signaling. These interconnected pathways demonstrate that HF development involves coordinated dysregulation of oxidative stress, energy metabolism, and cellular homeostasis, suggesting that therapeutic approaches targeting multiple nodes in this inflammatory-metabolic network may be most effective.

### 4.3. Clinical implications

The biological plausibility of our identified causal relationships is supported by substantial experimental evidence. Interferon gamma has been shown to activate STAT1 signaling pathway, leading to mitochondrial dysfunction and apoptosis through down-regulation of anti-apoptotic proteins like Bcl-2, which aligns with our finding of altered energy metabolites.^[42]^ MMP-1’s effects extend beyond matrix degradation to include activation of protease-activated receptors, explaining its broad metabolic associations.^[43]^ The protective effects of IL-10 and other anti-inflammatory factors operate through preservation of mitochondrial function and reduction of reactive oxygen species, consistent with our metabolomic findings.^[44,45]^ Recent studies have demonstrated that N-methyl-2-pyridone-5-carboxamide reflects NAD + depletion in failing hearts,^[46]^ while altered sphingolipid metabolism has been directly linked to cardiac hypertrophy and fibrosis.^[47]^ These established mechanisms provide strong biological support for our MR findings and suggest that the identified inflammatory-metabolic interactions represent genuine pathophysiological processes rather than statistical artifacts.

Our findings also have significant implications for biomarker development and clinical monitoring. The complex network of inflammatory factor-metabolite interactions suggests that multi-marker approaches might provide more comprehensive risk assessment than traditional single-marker strategies.^[48]^ For instance, combining measurements of interferon gamma with its associated metabolites (suberate and imidazole propionate) could improve risk prediction accuracy.^[49]^ The strong and consistent associations of certain metabolites, particularly N-methyl-2-pyridone-5-carboxamide, with HF risk suggest their potential utility as early warning markers.^[50,51]^ These biomarker combinations could enable more precise patient stratification and treatment monitoring, supporting the movement toward personalized medicine in HF management.^[52]^

The preventive implications of our findings are particularly noteworthy. Understanding the causal relationships between inflammatory factors, metabolites, and HF enables more targeted preventive strategies. The involvement of multiple metabolic pathways, including oxidative stress and lipid metabolism, suggests that comprehensive preventive approaches addressing multiple biological processes might be most effective.^[10]^ For instance, interventions targeting both inflammatory pathways and their associated metabolic alterations could provide more robust prevention strategies than single-pathway approaches.^[33,53]^

Implementing these findings in clinical practice requires careful consideration of several factors. The causal nature of the identified relationships suggests that early intervention might be crucial for optimal outcomes.^[54]^ Additionally, the complex network of interactions indicates that patient-specific inflammatory and metabolic profiles might necessitate tailored therapeutic approaches.^[55]^ While comprehensive profiling of multiple markers might be resource-intensive, strategic selection of key markers based on our findings could provide a cost-effective approach to risk assessment and treatment monitoring.^[33]^

Translation of these findings into clinical practice could significantly impact HF management across the spectrum from prevention to treatment. The integration of inflammatory and metabolic markers into clinical decision-making could enhance risk stratification, enable earlier intervention, and support more personalized treatment approaches.^[55]^ Moreover, the identification of multiple potential therapeutic targets suggests that combination therapies addressing both inflammatory and metabolic pathways might offer superior outcomes compared to current single-target approaches.

### 4.4. Strengths, limitations, and future directions

We acknowledge that horizontal pleiotropy was initially detected for several metabolites in our analysis, which could potentially bias causal inference. This occurs when genetic variants influence heart failure through pathways independent of the metabolite of interest. However, we systematically addressed this concern through rigorous methodological approaches. Using MR-PRESSO, we identified and removed outlier variants exhibiting pleiotropic effects, while additional sensitivity analyses including MR-Egger regression, weighted median, and weighted mode estimators confirmed the consistency of our findings after outlier removal. Importantly, our primary results remained stable following these corrections, suggesting that horizontal pleiotropy did not substantially influence our main conclusions about metabolite-heart failure relationships. While alternative genetic instruments derived from different populations or constructed based on biological pathway knowledge rather than purely statistical associations might further mitigate pleiotropy concerns in future studies, our comprehensive analytical approach effectively addressed these issues in the current investigation. This robust methodology provides confidence that the reported causal relationships represent true biological effects rather than artifacts of pleiotropy.

However, several limitations warrant consideration. An important limitation of our study is that, the MR approach relies on key assumptions that cannot be fully verified, and integrating GWAS datasets for metabolites and inflammatory markers from different sources may introduce batch effects and cross-cohort biases, which we attempted to mitigate through European ancestry population selection, strict quality control procedures, and sensitivity analyses, though future studies using data from the same cohort would be valuable for validation. While our metabolomics platform was extensive, some potentially important metabolites may not have been captured, and the dynamic nature of metabolic processes means that our measurements represent only a snapshot of these complex interactions. Another limitation of our study is the lack of validation in independent cohorts. However, our analysis already incorporates the largest publicly available GWAS datasets for heart failure, inflammatory factors, and metabolomics currently available globally. At present, there are no comparable alternative datasets with similar measurements and sufficient scale to enable meaningful independent validation while maintaining adequate statistical power. To address this limitation, we implemented multiple methodological approaches (IVW, MR-Egger, weighted median, and weighted mode) and extensive sensitivity analyses to internally validate our findings and ensure reproducibility.

These limitations suggest important directions for future research. Experimental validation of our findings, particularly the novel inflammatory factor-metabolite relationships, is crucial and could be pursued through cell culture and animal models. Longitudinal studies incorporating repeated measurements in diverse populations could help clarify the temporal dynamics of these relationships. Future validation and verification in independent cohorts are necessary to assess the robustness and generalizability of the findings in this study. Additionally, clinical translation research is needed to evaluate the practical utility of our findings, including the development of biomarker panels and therapeutic interventions targeting the identified pathways.

Clinical translation and establishing clinical thresholds are one of the future research directions, the causal framework provided in this study offers a scientifically rigorous foundation for designing the prospective studies needed to determine these clinical parameters. First, identifying causal biomarkers is a crucial first step toward developing clinical risk stratification tools. Our study provided a prioritized list of inflammatory factors and metabolites that warrant further investigation in prospective cohort studies to establish clinical cutoff values. The causal nature of these associations suggests that investments in developing clinical assays and establishing thresholds for these specific markers would be worthwhile. Second, our findings have immediate implications for therapeutic target prioritization. The identification of both protective (e.g., IL-10, CD40L receptor) and risk-promoting factors (e.g., interferon gamma, MMP-1) provides clear directions for drug development and repurposing efforts. For instance, therapies that enhance IL-10 signaling or inhibit interferon gamma pathways could be prioritized for clinical trials in HF prevention. Third, the metabolic signatures we identified, particularly N-methyl-2-pyridone-5-carboxamide and its relationship with inflammatory factors, could serve as pharmacodynamic biomarkers in clinical trials, helping to assess treatment response even before clinical thresholds are established.

## 5. Conclusion

Our comprehensive MR analysis has identified novel causal relationships in the inflammatory factor-metabolite-HF pathway. We found 9 inflammatory factors (out of 91) significantly associated with HF risk, including 3 risk-promoting factors (interferon gamma, MMP-1, and TNF-beta) and 6 protective factors (CD40L receptor, DNER, IL-10, LIFR, TNFSF12, and uPA). Additionally, we identified thirteen metabolites (out of 1400) robustly associated with HF risk, with N-methyl-2-pyridone-5-carboxamide showing the strongest evidence. Further investigation revealed 23 significant inflammatory factor-metabolite pairs (out of 1000s of possible combinations), highlighting potential mechanistic pathways. These findings elucidate the complex interplay between inflammation and metabolism in HF development, providing new insights for biomarker development and therapeutic strategies.

## Author contributions

**Conceptualization:** Xiuyan Guan, Yanping Wang.

**Data curation:** Xiuyan Guan, Yanping Wang.

**Formal analysis:** Xiuyan Guan.

**Investigation:** Xiuyan Guan.

**Supervision:** Yanping Wang.

**Validation:** Yanping Wang.

**Writing – original draft:** Xiuyan Guan.

**Writing – review & editing:** Yanping Wang.

## Supplementary Material



## References

[1] Khan MS Shahid I Bennis A Rakisheva A Metra M Butler J. Global epidemiology of heart failure. Nat Rev Cardiol. 2024;21:717–34. 38926611 10.1038/s41569-024-01046-6

[2] Zhang JJ Pogwizd SM Fukuda K. Trials and tribulations of cell therapy for heart failure: an update on ongoing trials. Nat Rev Cardiol. 2024;22:372–85. 39548233 10.1038/s41569-024-01098-8

[3] Cook C Cole G Asaria P Jabbour R Francis DP. The annual global economic burden of heart failure. Int J Cardiol. 2014;171:368–76. 24398230 10.1016/j.ijcard.2013.12.028

[4] Li Z Zhao H Wang J. Metabolism and chronic inflammation: the links between chronic heart failure and comorbidities. Front Cardiovasc Med. 2021;8:650278. 34026868 10.3389/fcvm.2021.650278 PMC8131678

[5] Ajoolabady A Pratico D Vinciguerra M Lip GYH Franceschi C Ren J. Inflammaging: mechanisms and role in the cardiac and vasculature. Trends Endocrinol Metab. 2023;34:373–87. 37076375 10.1016/j.tem.2023.03.005

[6] Huang L Shen R Yu H. The levels of systemic inflammatory markers exhibit a positive correlation with the occurrence of heart failure: a cross-sectional study from NHANES. Front Cardiovasc Med. 2024;11:1457534. 39465132 10.3389/fcvm.2024.1457534 PMC11502476

[7] Karwi QG Uddin GM Ho KL Lopaschuk GD. Loss of metabolic flexibility in the failing heart. Front Cardiovasc Med. 2018;5:68. 29928647 10.3389/fcvm.2018.00068 PMC5997788

[8] Bertero E Maack C. Metabolic remodelling in heart failure. Nat Rev Cardiol. 2018;15:457–70. 29915254 10.1038/s41569-018-0044-6

[9] Hunter WG Kelly JP McGarrah RW 3rd Kraus WE Shah SH. Metabolic dysfunction in heart failure: diagnostic, prognostic, and pathophysiologic insights from metabolomic profiling. Curr Heart Fail Rep. 2016;13:119–31. 27216948 10.1007/s11897-016-0289-5 PMC5504685

[10] Neves LS Saraiva F Ferreira R Leite-Moreira A Barros AS Diaz SO. Metabolomics and cardiovascular risk in patients with heart failure: a systematic review and meta-analysis. Int J Mol Sci. 2024;25:5693. 38891881 10.3390/ijms25115693 PMC11172189

[11] Arumugam S Sreedhar R Thandavarayan RA Karuppagounder V Watanabe K. Targeting fatty acid metabolism in heart failure: is it a suitable therapeutic approach? Drug Discov Today. 2016;21:1003–8. 26905600 10.1016/j.drudis.2016.02.010

[12] Zheng J Baird D Borges M-C. Recent developments in Mendelian randomization studies. Current Epidemiology Reports. 2017;4:330–45. 29226067 10.1007/s40471-017-0128-6 PMC5711966

[13] Huang X Hu L Li J Tao S Xue T. The relationship between inflammatory factors and heart failure: evidence based on bidirectional Mendelian randomization analysis. Front Cardiovasc Med. 2024;11:1378327. 39726944 10.3389/fcvm.2024.1378327 PMC11669679

[14] Virak V Nov P Chen D. Exploring the impact of metabolites function on heart failure and coronary heart disease: insights from a Mendelian randomization (MR) study. Am J Cardiovasc Dis. 2024;14:242–54. 39309113 10.62347/OQXZ7740 PMC11410790

[15] Zhu XG Liu GQ Peng YP. Causal correlations between inflammatory proteins and heart failure: a two-sample Mendelian randomization analysis. ESC Heart Fail. 2024;12:1374–85. 39501838 10.1002/ehf2.15151 PMC11911586

[16] Purcell S Neale B Todd-Brown K. PLINK: a tool set for whole-genome association and population-based linkage analyses. Am J Hum Genet. 2007;81:559–75. 17701901 10.1086/519795 PMC1950838

[17] Fairley S Lowy-Gallego E Perry E Flicek P. The International Genome Sample Resource (IGSR) collection of open human genomic variation resources. Nucleic Acids Res. 2020;48:D941–7. 31584097 10.1093/nar/gkz836 PMC6943028

[18] Xue H Shen X Pan W. Constrained maximum likelihood-based Mendelian randomization robust to both correlated and uncorrelated pleiotropic effects. Am J Hum Genet. 2021;108:1251–69. 34214446 10.1016/j.ajhg.2021.05.014 PMC8322939

[19] Pierce BL Ahsan H Vanderweele TJ. Power and instrument strength requirements for Mendelian randomization studies using multiple genetic variants. Int J Epidemiol. 2011;40:740–52. 20813862 10.1093/ije/dyq151 PMC3147064

[20] Bowden J Smith GD Burgess S. Mendelian randomization with invalid instruments: effect estimation and bias detection through Egger regression. Int J Epidemiol. 2015;44:512–25. 26050253 10.1093/ije/dyv080 PMC4469799

[21] Bowden J Smith GD Haycock PC Burgess S. Consistent estimation in Mendelian randomization with some invalid instruments using a weighted median estimator. Genet Epidemiol. 2016;40:304–14. 27061298 10.1002/gepi.21965 PMC4849733

[22] Lee CH Cook S Lee JS Han B. Comparison of two meta-analysis methods: inverse-variance-weighted average and weighted sum of Z-Scores. Genomics Inform. 2016;14:173–80. 28154508 10.5808/GI.2016.14.4.173 PMC5287121

[23] Burgess S Bowden J Fall T Ingelsson E Thompson SG. Sensitivity analyses for robust causal inference from Mendelian randomization analyses with multiple genetic variants. Epidemiology. 2017;28:30–42. 27749700 10.1097/EDE.0000000000000559 PMC5133381

[24] Burgess S Thompson SG. Interpreting findings from Mendelian randomization using the MR-Egger method. Eur J Epidemiol. 2017;32:377–89. 28527048 10.1007/s10654-017-0255-x PMC5506233

[25] Verbanck M Chen CY Neale B Do R. Detection of widespread horizontal pleiotropy in causal relationships inferred from Mendelian randomization between complex traits and diseases. Nat Genet. 2018;50:693–8. 29686387 10.1038/s41588-018-0099-7 PMC6083837

[26] Bowden J Del Greco MF Minelli C Smith GD Sheehan N Thompson J. A framework for the investigation of pleiotropy in two-sample summary data Mendelian randomization. Stat Med. 2017;36:1783–802. 28114746 10.1002/sim.7221 PMC5434863

[27] Skrivankova VW Richmond RC Woolf BAR. Strengthening the reporting of observational studies in epidemiology using Mendelian randomization: the STROBE-MR statement. JAMA. 2021;326:1614–21. 34698778 10.1001/jama.2021.18236

[28] Daub S Lutgens E Münzel T Daiber A. CD40/CD40L and related signaling pathways in cardiovascular health and disease-the Pros and Cons for cardioprotection. Int J Mol Sci . 2020;21:8533. 33198327 10.3390/ijms21228533 PMC7697597

[29] Smolgovsky S Ibeh U Tamayo TP Alcaide P. Adding insult to injury - Inflammation at the heart of cardiac fibrosis. Cell Signal. 2021;77:109828. 33166625 10.1016/j.cellsig.2020.109828 PMC7718304

[30] MacGrogan D Münch J de la Pompa JL. Notch and interacting signalling pathways in cardiac development, disease, and regeneration. Nat Rev Cardiol. 2018;15:685–704. 30287945 10.1038/s41569-018-0100-2

[31] Kühn R Löhler J Rennick D Rajewsky K Müller W. Interleukin-10-deficient mice develop chronic enterocolitis. Cell. 1993;75:263–74. 8402911 10.1016/0092-8674(93)80068-p

[32] York AG Skadow MH Oh J. IL-10 constrains sphingolipid metabolism to limit inflammation. Nature. 2024;627:628–35. 38383790 10.1038/s41586-024-07098-5 PMC10954550

[33] Zheng Z Tan X. Mendelian randomization of plasma lipidome, inflammatory proteome and heart failure. ESC Heart Fail. 2024;11:4209–21. 39145416 10.1002/ehf2.14997 PMC11631237

[34] Saraiva M Vieira P O’Garra A. Biology and therapeutic potential of interleukin-10. J Exp Med. 2020;217:e20190418. 31611251 10.1084/jem.20190418 PMC7037253

[35] Reifenberg K Lehr H-A Torzewski M. Interferon-γ induces chronic active myocarditis and cardiomyopathy in transgenic mice. Am J Pathol. 2007;171:463–72. 17556594 10.2353/ajpath.2007.060906 PMC1934522

[36] Chiang FF Chao TH Huang SC Cheng CH Tseng YY Huang YC. Cysteine regulates oxidative stress and glutathione-related antioxidative capacity before and after colorectal tumor resection. Int J Mol Sci. 2022;23:9581. 36076975 10.3390/ijms23179581 PMC9455234

[37] Toprak G Yüksel H Demirpençe O Islamoglu Y Evliyaoglu O Mete N. Fibrosis in heart failure subtypes. Eur Rev Med Pharmacol Sci. 2013;17:2302–9. 24065222

[38] Nagase H Visse R Murphy G. Structure and function of matrix metalloproteinases and TIMPs. Cardiovasc Res. 2006;69:562–73. 16405877 10.1016/j.cardiores.2005.12.002

[39] DeLeon-Pennell KY Meschiari CA Jung M Lindsey ML. Matrix metalloproteinases in myocardial infarction and heart failure. Prog Mol Biol Transl Sci. 2017;147:75–100. 28413032 10.1016/bs.pmbts.2017.02.001 PMC5576003

[40] Naoum JJ Chai H Lin PH Lumsden AB Yao Q Chen C. Lymphotoxin-alpha and cardiovascular disease: clinical association and pathogenic mechanisms. Med Sci Monit. 2006;12:RA121–4. 16810143

[41] Wróbel-Nowicka K Wojciechowska C Jacheć W Zalewska M Romuk E. The role of oxidative stress and inflammatory parameters in heart failure. Medicina (Kaunas, Lithuania). 2024;60:760. 38792942 10.3390/medicina60050760 PMC11123446

[42] Cao ZH Zheng QY Li GQ. STAT1-mediated down-regulation of Bcl-2 expression is involved in IFN-γ/TNF-α-induced apoptosis in NIT-1 cells. PLoS One. 2015;10:e0120921. 25811609 10.1371/journal.pone.0120921 PMC4374929

[43] Pei D. Matrix metalloproteinases target protease-activated receptors on the tumor cell surface. Cancer Cell. 2005;7:207–8. 15766657 10.1016/j.ccr.2005.02.011

[44] Dokka S Shi X Leonard S Wang L Castranova V Rojanasakul Y. Interleukin-10-mediated inhibition of free radical generation in macrophages. Am J Physiol Lung Cell Mol Physiol. 2001;280:L1196–202. 11350798 10.1152/ajplung.2001.280.6.L1196

[45] Ip WKE Hoshi N Shouval DS Snapper S Medzhitov R. Anti-inflammatory effect of IL-10 mediated by metabolic reprogramming of macrophages. Science. 2017;356:513–9. 28473584 10.1126/science.aal3535 PMC6260791

[46] Blanco-Vaca F Rotllan N Canyelles M Mauricio D Escolà-Gil JC Julve J. NAD+-increasing strategies to improve cardiometabolic health? Front Endocrinol (Lausanne). 2021;12:815565. 35173682 10.3389/fendo.2021.815565 PMC8842632

[47] Qu Y Wang Y Wu T Liu X Wang H Ma D. A comprehensive multiomics approach reveals that high levels of sphingolipids in cardiac cachexia adipose tissue are associated with inflammatory and fibrotic changes. Lipids Health Dis. 2023;22:211. 38041133 10.1186/s12944-023-01967-0 PMC10691093

[48] Adamcova M Šimko F. Multiplex biomarker approach to cardiovascular diseases. Acta Pharmacol Sin. 2018;39:1068–72. 29645001 10.1038/aps.2018.29 PMC6289323

[49] Raju SC Molinaro A Awoyemi A. Microbial-derived imidazole propionate links the heart failure-associated microbiome alterations to disease severity. Genome Med. 2024;16:27. 38331891 10.1186/s13073-024-01296-6 PMC10854170

[50] Ferrell M Wang Z Anderson JT. A terminal metabolite of niacin promotes vascular inflammation and contributes to cardiovascular disease risk. Nat Med. 2024;30:424–34. 38374343 10.1038/s41591-023-02793-8 PMC11841810

[51] Walker MA Tian R. NAD metabolism and heart failure: mechanisms and therapeutic potentials. J Mol Cell Cardiol. 2024;195:45–54. 39096536 10.1016/j.yjmcc.2024.07.008 PMC11390314

[52] Castiglione V Aimo A Vergaro G Saccaro L Passino C Emdin M. Biomarkers for the diagnosis and management of heart failure. Heart Fail Rev. 2022;27:625–43. 33852110 10.1007/s10741-021-10105-w PMC8898236

[53] Remmelzwaal S van Oort S Handoko ML van Empel V Heymans SRB Beulens JWJ. Inflammation and heart failure: a two-sample Mendelian randomization study. J Cardiovasc Med (Hagerstown). 2022;23:728–35. 36166332 10.2459/JCM.0000000000001373

[54] Xiao N Nie M Pang H. Integrated cytokine and metabolite analysis reveals immunometabolic reprogramming in COVID-19 patients with therapeutic implications. Nat Commun. 2021;12:1618. 33712622 10.1038/s41467-021-21907-9 PMC7955129

[55] Metwaly A Reitmeier S Haller D. Microbiome risk profiles as biomarkers for inflammatory and metabolic disorders. Nature Rev Gastroenterol Hepatol. 2022;19:383–97. 35190727 10.1038/s41575-022-00581-2

