# Supplementary material for: Dissecting causal networks of inflammatory factors and metabolites in heart failure: A mediation Mendelian randomization study
Source: Medicine (Baltimore). 2025 Aug 8;104(32):e43801. doi: 10.1097/MD.0000000000043801 (PMC12338296; doi:10.1097/MD.0000000000043801)
Supplement: Supplementary file 1 [file medi-104-e43801-s001.docx]

**Supplementary Table S1.** Mendelian Randomization Assumptions and Testing Methods.

| **MR Assumption** | **Description** | **Testing Method** | **Implementation** |
| --- | --- | --- | --- |
| Relevance | Genetic instruments strongly associated with exposure | F-statistic | F > 10 for all included instruments |
| Independence | Genetic instruments not associated with confounders | LD analysis; Pleiotropy assessment | PLINK 1.9 software; Physical distance threshold 10,000kb; LD coefficient R² threshold 0.001; MR-Egger intercept test |
| Exclusion Restriction | Genetic instruments affect outcome only through exposure | Multiple MR methods; Heterogeneity and outlier detection | IVW (primary method), MR-Egger, weighted median, weighted mode; MR-PRESSO global test; Cochran's Q test; Leave-one-out analysis |
